# Breast Cancer with Bone Metastasis: Molecular Insights and Clinical Management

**DOI:** 10.3390/cells10061377

**Published:** 2021-06-02

**Authors:** Konstantinos Venetis, Roberto Piciotti, Elham Sajjadi, Marco Invernizzi, Stefania Morganti, Carmen Criscitiello, Nicola Fusco

**Affiliations:** 1 Department of Oncology and Hemato-Oncology, University of Milan, 20141 Milan, Italy; konstantinos.venetis@unimi.it (K.V.); roberto.piciotti@unimi.it (R.P.); elham.sajjadi@unimi.it (E.S.); stefania.morganti@ieo.it (S.M.); 2 Division of Pathology, IEO, European Institute of Oncology IRCCS, 20141 Milan, Italy; 3 Department of Health Sciences, University of Eastern Piedmont, 28100 Novara, Italy; marco.invernizzi@med.uniupo.it; 4 Infrastruttura Ricerca Formazione Innovazione (IRFI), Azienda Ospedaliera SS. Antonio e Biagio e Cesare Arrigo, 15121 Alessandria, Italy; 5 Division of Early Drug Development for Innovative Therapies, IEO, European Institute of Oncology IRCCS, 20141 Milan, Italy

**Keywords:** breast cancer, bone metastasis, therapy resistance, tumor progression, tumor-bone microenvironment, bone-targeting therapy

## Abstract

Despite the remarkable advances in the diagnosis and treatment of breast cancer patients, the presence or development of metastasis remains an incurable condition. Bone is one of the most frequent sites of distant dissemination and negatively impacts on patient’s survival and overall frailty. The interplay between tumor cells and the bone microenvironment induces bone destruction and tumor progression. To date, the clinical management of bone metastatic breast cancer encompasses anti-tumor systemic therapies along with bone-targeting agents, aimed at slowing bone resorption to reduce the risk of skeletal-related events. However, their effect on patients’ survival remains controversial. Unraveling the biology that governs the interplay between breast neoplastic cells and bone tissue would provide means for the development of new therapeutic agents. This article outlines the state-of-the art in the characterization and targeting the bone metastasis in breast cancer, focusing on the major clinical and translational studies on this clinically relevant topic.

## 1. Introduction

Breast cancer is the most prevalent malignancy and the foremost cause of cancer-related death in women worldwide [1]. Despite the achievements in the management of this tumor, breast cancer remains an incurable disease when it is diagnosed, or it has progressed, towards advanced stages [2]. Hence, the median overall survival (OS) of patients with metastatic breast cancer (MBC) ranges from 2 to 3 years, with a 27% overall 5-year relative survival rate [3]. The most common sites of distant metastasis include bones, lungs, liver, and brain [4]. Among these, the bone is affected in more than 70% of patients with MBC [5,6,7].

Bone metastases not only considerably reduce the OS but also the health-related quality of life due to pain, fatigue, and skeletal-related events (SREs) [8,9,10]. Several therapeutic strategies to specifically target this condition (e.g., bone-modifying agents) are currently available [9,11,12,13]. However, their reliability and impact on patients’ frailty remain a subject of debate [14]. This could be due to the lack of a complete understanding of the crosstalk between breast cancer circulating cells, tumor microenvironment, muscle tissue, and bone microenvironment [15,16,17,18,19]. Improved clinical management of patients with MBC to the bone not only requires an appropriate combination of systemic and bone-targeting agents, but also the precise identification of highly responsive patients using a precision medicine approach.

In this review, we provide an overview of the biological models and the molecular heterogeneity that characterizes bone metastasis in breast cancer. Emphasis is also placed on the currently available systemic therapies and bone-modifying agents through a broad overview of the main trials involving patients with breast cancer and bone metastasis, in order to highlight the current and future therapeutic implications.

## 2. Biological Mechanisms of Bone Metastasis

**Figure 1:**
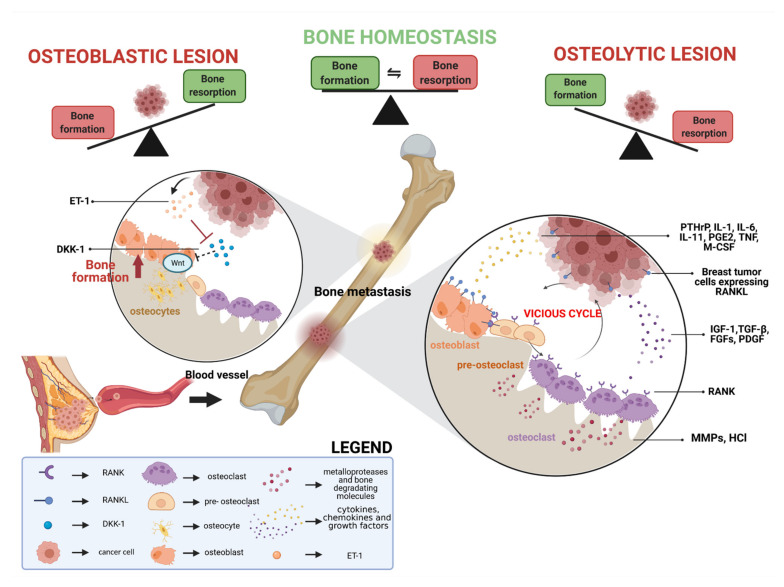
Schematic representation of the processes involved in breast cancer bone metastasis formation. Metastatic tumor cells migrate from the breast primary site to the bone through the bloodstream. Once they arrive in the target part of the skeleton, these neoplastic clones are able to activate a cascade of events that lead to a biological vicious cycle, ultimately leading to the dysregulation of the normal bone homeostasis. In particular, breast cancer bone metastasis can be either osteolytic or osteoblastic based on the type of mechanism that prevails in the bone disequilibrium (i.e., bone resorption or formation). When osteoclastogenic pathways are activated by the metastatic clones, several trophic factors, cytokines, and chemokines (e.g., PTHrP, IL1, IL6, IL11, PGE2, TNF and M-CSF) are secreted. These, either directly or indirectly (via osteoblasts), stimulate osteoclast differentiation and activity through a vicious cycle. Moreover, RANKL produced by both breast cancer cells and osteoblasts binds on RANK receptors, further stimulating the differentiation of the osteoclasts. These events lead to enhanced bone resorption and consequent release of metalloproteases, HCl and matrix-embedded growth factors (e.g., IGF-1, TGF-β, FGF and PDGF), which in turn cause breakdown of the collagenous matrix and promote cancer cell proliferation and tumor progression, respectively. In osteoblastic lesions, ET1 secreted by breast cancer cells inhibits the expression of DKK-1, which normally blocks Wnt signaling decreasing osteoblastic differentiation. Inhibition of DKK-1 results in an increased osteoblast activity favoring uncontrolled bone formation. Abbreviations: PTHrP, parathyroid hormone-related peptide; IL1, interleukin 1; IL6, interleukin 6; IL11, interleukin 11; PGE2, prostaglandin E2; TNF, tumor necrosis factor; M-CSF, macrophage colony-stimulating factor; HCl, hydrochloric acid; IGF1, insulin-growth factor 1; TGFβ, transforming growth factor β; FGF, fibroblast growth factor; PDGF, platelet-derived growth factor. ET1, endothelin 1; DKK1, Dickkopf 1.

The metastatic process is defined as the dissemination of neoplastic cells from the primary neoplasm to secondary sites [4]. Based upon a radiologic assessment that demonstrates bone destruction or deposition of new bone tissue, bone metastases are classified as osteolytic, osteoblastic, or mixed [20]. Although breast cancer bone metastases are predominantly osteolytic, 15–20% of cases have a predominant osteoblastic component [21,22,23,24]. In normal conditions, several bone modifications occur within the physiological process of bone remodeling [6,25]. When the rate of bone resorption exceeds osteogenesis, bone density decreases but remains close to normal levels [26,27]. Unbalances in this mechanism lead to an increased risk of fractures, particularly at the distal femur and proximal tibia levels [28]. This complex process is regulated by resident bone cells and other cell types of the bone microenvironment, including lymphocytes, macrophages, hematopoietic cells, and endocrine signaling molecules [29,30,31,32,33,34]. In particular, the discovery of endocrine mediators produced by the skeleton has radically changed our understanding not only of the bone biology but also of the endocrinology in general [34]. Given the intrinsic nature of the bone (i.e., hard tissue composed of a mineralized matrix), however, the invasion of cancer cells is naturally difficult in this tissue [35]. This characteristic is in apparent contradiction with the high frequency of bone metastases in breast cancer. However, there is an intricate network of pathways that enhances the potential for breast cancer metastatic clones to invade the bone (Figure 1).

In osteolytic lesions, osteoclast-mediated bone resorption is a key and early step [36]. The interaction between the receptor activator of nuclear factor-kappa B (RANK) and its ligand (RANKL) plays a consistent part in this process [21,37,38]. Specifically, increased RANKL levels lead to hyperactivation of osteoclastogenesis and bone resorption, paving the way for metastatic clones to invade the bone [39,40]. Osteoblasts and osteoclasts secrete a series of trophic factors, cytokines, and chemokines, initiating the vicious cycle that promotes bone destruction and tumor progression [41,42]. In this regard, parathyroid hormone-related peptide (PTHrP), interleukin (IL)1, IL6, IL11, prostaglandin E2 (PGE2), tumor necrosis factor (TNF), and macrophage colony-stimulating factor (M-CSF) are released in the bone microenvironment promoting differentiation of the osteoblasts [40,43,44,45,46,47,48,49]. Moreover, RANKL produced by both breast cancer cells and osteoblast further stimulates osteoclast differentiation and activity-binding RANK on the cell surface [50,51]. In contrast, osteoblasts secrete osteoprotegerin (OPG), a soluble decoy receptor for RANKL, which inhibits RANK/RANKL signaling negatively regulating osteoclastogenesis [52,53,54]. Of note, osteoclast differentiation may also be elicited by IL6, IL1, prostaglandins, and M-CSF-mediated stimulation of bone marrow macrophages [25,55,56]. Upon activation, osteoclasts reabsorb the bone by producing hydrochloric acid and metalloproteases, which dissolve the mineral in bone and cause the breakdown of the collagenous matrix, respectively [57]. Bone reabsorption causes the release of various growth factors that are stored in the bone matrix including insulin-growth factor 1 (IGF1), transforming growth factor β (TGF-β), fibroblast growth factor (FGFs) and platelet-derived growth factor (PDGF) [58,59,60]. Among these, IGF1 activates phosphoinositide 3-kinase (PI3K)/Akt mammalian target of rapamycin (mTOR) pathway, with subsequent breast cancer cell growth, proliferation, and migration into the bone [17,61,62]. Osteoblasts can also be regulated by metastatic tumor cell-derived factors including endothelin 1 (ET1), dickkopf 1 (DKK1), and the Wnt signaling cascade [36,63,64,65,66]. While Wnt promotes osteoblast differentiation, its activity can be inhibited by DKK1, which is an antagonist in this cascade. ET1 downregulates the expression of DKK1, which allows the activation of Wnt inducing an osteoblastic phenotype in breast cancer bone metastases [57]. Despite these insights, further elucidation of this biological pattern is needed.

## 3. Bone-Targeting Therapies for Breast Cancer Patients with Bone Metastasis

Patients with breast cancer metastatic to the bone require a multidisciplinary approach that should consider not only the clinical scenario but also the tumor specific biology [9,18,67,68,69,70,71,72]. Indeed, the metastatic process involves several pathways that are intimately related to breast cancer biomarkers, such as estrogen receptor, progesterone receptor, and HER2 [73]. Not surprisingly, these intrinsic characteristics govern the tailored treatment in patients with breast cancer. The currently available therapeutic strategies include a combination of the systemic therapies used in breast cancer (e.g., chemotherapy, ET, radiotherapy) and those specifically targeting the bone, known as bone-modifying agents [36]. These drugs aim at inhibiting the activity of osteoclasts, thereby decelerating the process of bone resorption [74]. Currently, bisphosphonates and RANK/RANKL inhibitors represent the foremost agents for the clinical management of patients with bone metastasis [75]. Emerging drugs include cathepsin K inhibitors, Src inhibitors, TGFβ blockers, C-X-C motif chemokine receptor type 4 (CXCR4) inhibitors, and αvβ3 integrin antagonists [39]. Current and forthcoming therapies are discussed below, and the corresponding clinical trials are summarized in Table 1.

Bisphosphonates have a dual role in decreasing bone resorption by exerting an apoptotic effect on osteoclasts and increasing mineralization by inhibiting osteoclast activity [76]. First-generation non-nitrogen-containing bisphosphonates (e.g., etidronate and clodronate) are metabolized intracellularly to analogs of ATP. These metabolites prevent bone resorption by inducing osteoclast apoptosis through the inhibition of ATP-dependent enzymes [77]. Conversely, next generation nitrogen-containing bisphosphonates (e.g., alendronate, ibandronate, pamidronate, risedronate and zoledronic acid) promote osteoclast apoptosis by inhibiting farnesyl pyrophosphate synthase (FPPS) and are considered more potent osteoclast inhibitors [78]. The administration of these agents may reduce the risk of SREs and skeletal morbidity rate. The phase III ZOOM trial (NCT00375427) evaluated the efficacy and safety of a reduced dosing frequency of zoledronic acid in 425 patients with breast cancer who had one or more bone metastases, and showed that the drug maintains its therapeutic effects [79]. Accordingly, the skeletal morbidity rate was 0.26 (95% confidence index [CI] 0.15–0.37) in patients treated with zoledronic acid every 12 weeks, versus 0.22 (0.14–0.29) in those treated once every 4 weeks [79]. Moreover, a randomized phase III trial including 855 patients with bone MBC found no increased risk of skeletal events over 2 years in patients who received zoledronic acid every 12 weeks compared with the standard dosing interval of every 4 weeks, suggesting that this longer interval may be an acceptable treatment option (NCT00869206) [80]. A recent meta-analysis that included 44 randomized trials involving 37,302 women with breast cancer at different disease stages assessed the effects of bisphosphonates on anti-cancer treatment [81]. Regarding breast cancer patients with bone metastasis, either intravenous or oral administration of bisphosphonates significantly reduced the absolute risk of SREs by 14% (RR 0.86, 95% CI 0.78–0.95) when compared with placebo [81]. Of note, bisphosphonates delayed the median time to an SRE and reduced bone pain in comparison with placebo or no bisphosphonate; however, no significant effect was observed in terms of overall survival.

The RANK/RANKL interaction significantly affects the progression of the deleterious vicious cycle between circulating breast cancer cells and the bone microenvironment. Therapeutic approaches targeting these molecules mainly rely on denosumab, a fully human monoclonal anti-RANKL antibody. This drug inhibits the RANKL/RANK signaling-mediated bone resorption, suppressing bone turnover and leading to the reduction of SRE risk [82,83]. Clinical trials that directly compared denosumab with zoledronic acid, demonstrated that the former was superior in terms of reducing bone turnover and pain as well as preventing SREs (NCT00321464) [84,85]. However, no significant differences were observed in overall survival and disease progression. Regrettably, some trials that assessed the effect of denosumab in bone metastatic breast cancer patients have been terminated without providing any essential insights (NCT03070002, NCT01952054) Notably, a novel orally available small-molecule RANKL inhibitor, AS2676293 has been found to markedly inhibit bone metastasis of human breast cancer cells in mouse models, possibly providing a more efficacious and affordable solution [86].

Additional therapeutic targets with potential clinical utility in the treatment of bone MBC are still under investigation. In this regard, odanacatib is an antagonist of cathepsin K, a protease produced by osteoclasts directly involved in bone resorption [87]. Although a phase II clinical trial carried out in bone MBC patients correlated odanacatib with reduced bone turnover and good toleration scores (i.e., if treatment-related dose-limiting toxicity was observed in ≤10 of 30 patients, odanacatib 5 mg was considered well-tolerated) (NCT00399802) [88], a phase III trial using odanacatib was withdrawn before subject enrolment (NCT00692458). Nevertheless, it has been recently reported that treatment with this agent is associated with an increased risk of stroke [89]. Moreover, dasatinib is an inhibitor of Src, a member of the nonreceptor tyrosine kinase family, which is overexpressed in breast cancer tissue positively regulating osteoclasts and negatively regulating osteoblasts [90]. A recently concluded phase I/II study showed that a combination of dasatinib with zoledronic acid presented clinical efficacy in treating breast cancer patients with bone metastasis (NCT00566618) [91]. Conversely, in a bone MBC population unselected by molecular markers, dasatinib did not improve the progression-free survival (PFS), stressing the need for implementation of molecularly-defined patient cohorts (NCT00410813) [92]. Finally, TGFβ, CXCR4, and αvβ3 integrin are key mediators of breast cancer metastasis to bone (Table 2) [93,94,95,96]. Antagonists of these proteins are under investigation in preclinical models of metastatic breast cancer, showing accumulating positive data [97,98,99,100,101,102,103,104,105,106,107,108]. Assessment of their safety and efficacy in phase I clinical trials is expected.

## 4. Concluding Remarks

An improved understanding of the mechanisms that drive the metastatic dissemination of breast cancer to the bone and the development of therapy resistance are essential to concretely establish the most suitable clinical management strategies for these patients. Pathways leading to bone remodeling represent the ideal target for future translational and clinical research studies. These discoveries would lead to a possible improvement in the precision medicine approach in the treatment of breast cancer with bone metastasis, not only for patients’ survival but also for their health-related quality of life. Novel agents including bisphosphonates and denosumab may now be administered along with the traditional therapeutic regimens. Despite the positive results of these drugs in reducing the risk of SREs, no significant effect has been observed in terms of OS. Future research should focus on a deeper understanding of metastatic heterogeneity as well as on identifying key regulators of molecular signaling pathways, which might be potential therapeutic targets.

## Figures and Tables

**Table 1:** Bone-modifying agents and corresponding clinical trials in patients with breast cancer metastatic to the bone. Abbreviations: C, completed; T, terminated; W, withdrawn; SMR, skeletal morbidity rate; SRE, skeletal-related events; PIS, pain intensity score; CTC, circulating tumor cells; PFS, progression-free survival; EMT, epithelial-mesenchymal transition; AEs, adverse events; u-NTx, urinary *n*-telopeptide of type I collagen; u-DPD, urinary deoxypyridinoline; MTD, maximum tolerated dose; DFS, disease-free survival; RR, response rate; Information has been obtained from clinicaltrials.gov and clinicaltrialsregister.eu.

Drug Class	Drug Name	Phase	Status	Patients	Basket Trial	Primary Outcome	Secondary Outcome	Trial Number
Bisphosphonates	Zoledronic acid	III	C	425	No	SMR	Incidence and proportion of SRE, Safety	NCT00375427
	Zoledronic acid	III	C	1822	Yes	SRE	PIS, Osteonecrosis of the jaw, renal dysfunction, SMR	NCT00869206
Monoclonal antibody	Denosumab vs. Zoledronic acid	III	C	2049	No	SRE	SRE	NCT00321464
	Denosumab	II	T (Low accrual)	1	No	Patients with reduced CTCs	Change in CTC, PFS	NCT03070002
	Denosumab	II	T	7	No	Effect in reducing CTCs	Changes of EMT in CTCs	NCT01952054
Cathepsin inhibitor	Odanacatib	II	C	43	No	Change in u-NTx, AEs	Change in u-DPD	NCT00399802
Src inhibitor	Dasatinib + Zoledronic acid	I/II	C	31	No	MTD	RR	NCT00566618
	Dasatinib	II	C	85	No	PFS	RR, MUC-1 Antigen Response, CTC RR	NCT00410813

**Table 2:** Antagonists of breast cancer bone metastasis mediators investigated in preclinical models.

Compounds	Mechanism of Action	References
LY2109761	TGFβ I and II dual inhibitor	[98,99,100,101]
AMD3465, AMD3100, AMD070	CXCR4 antagonists	[102,103,104,105]
ProAgio	integrin alphavbeta3 inhibitor	[106,107,108]

## Data Availability

Not applicable.
